# Glypicans and Heparan Sulfate in Synaptic Development, Neural Plasticity, and Neurological Disorders

**DOI:** 10.3389/fncir.2021.595596

**Published:** 2021-02-10

**Authors:** Keisuke Kamimura, Nobuaki Maeda

**Affiliations:** Developmental Neuroscience Project, Department of Brain and Neurosciences, Tokyo Metropolitan Institute of Medical Science, Setagaya, Japan

**Keywords:** glypican, heparan sulfate proteoglycan, neurexin, synapse-organizing protein, synaptic plasticity, autism spectrum disorder, schizophrenia

## Abstract

Heparan sulfate proteoglycans (HSPGs) are components of the cell surface and extracellular matrix, which bear long polysaccharides called heparan sulfate (HS) attached to the core proteins. HSPGs interact with a variety of ligand proteins through the HS chains, and mutations in HSPG-related genes influence many biological processes and cause various diseases. In particular, recent findings from vertebrate and invertebrate studies have raised the importance of glycosylphosphatidylinositol-anchored HSPGs, glypicans, as central players in the development and functions of synapses. Glypicans are important components of the synapse-organizing protein complexes and serve as ligands for leucine-rich repeat transmembrane neuronal proteins (LRRTMs), leukocyte common antigen-related (LAR) family receptor protein tyrosine phosphatases (RPTPs), and G-protein-coupled receptor 158 (GPR158), regulating synapse formation. Many of these interactions are mediated by the HS chains of glypicans. Neurexins (Nrxs) are also synthesized as HSPGs and bind to some ligands in common with glypicans through HS chains. Therefore, glypicans and Nrxs may act competitively at the synapses. Furthermore, glypicans regulate the postsynaptic expression levels of ionotropic glutamate receptors, controlling the electrophysiological properties and non-canonical BMP signaling of synapses. Dysfunctions of glypicans lead to failures in neuronal network formation, malfunction of synapses, and abnormal behaviors that are characteristic of neurodevelopmental disorders. Recent human genetics revealed that glypicans and HS are associated with autism spectrum disorder, neuroticism, and schizophrenia. In this review, we introduce the studies showing the roles of glypicans and HS in synapse formation, neural plasticity, and neurological disorders, especially focusing on the mouse and *Drosophila* as potential models for human diseases.

## Introduction

**FIGURE 1:**
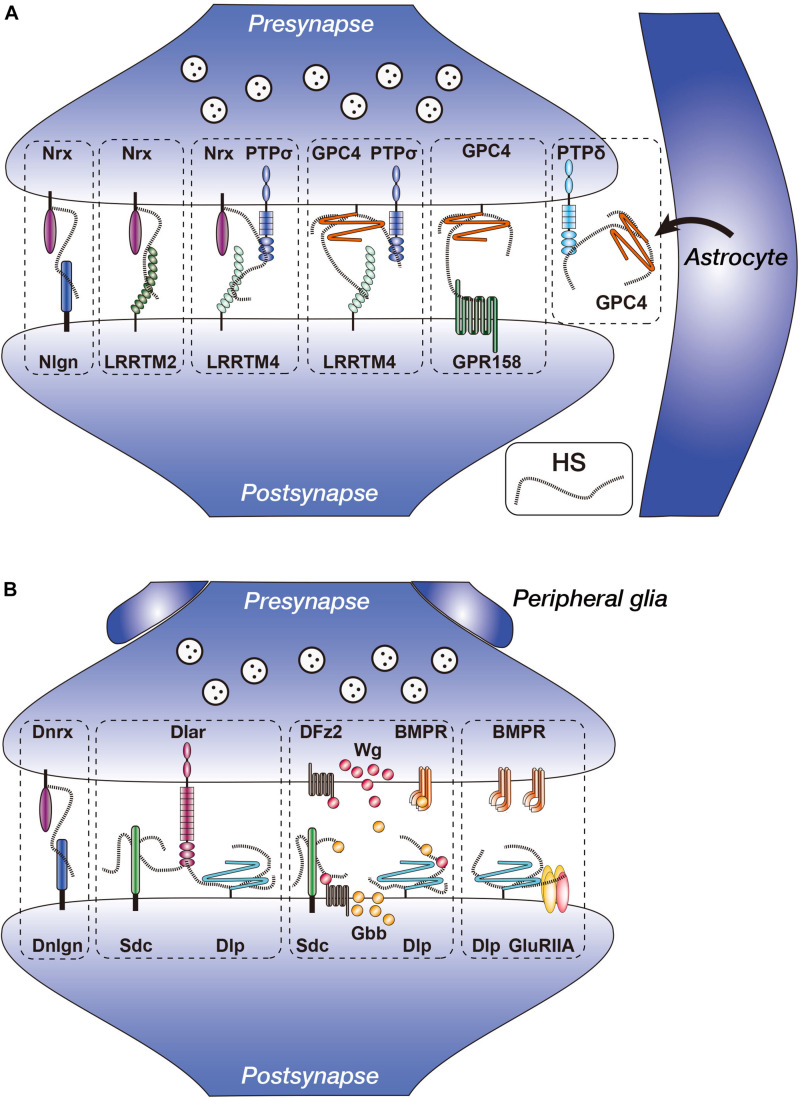
Vertebrate and *Drosophila* heparan sulfate proteoglycans (HSPGs) in the synapses. **(A)** Heparan sulfate (HS) chains of Nrx and GPC4 interact with multiple synapse-organizing proteins. Presynaptic Nrx interacts with Nlgn, LRRTM2, LRRTM4, and PTPσ. Presynaptic GPC4 interacts with LRRTM4, PTPσ, and GPR158. Nrx–PTPσ–LRRTM4 and GPC4–PTPσ–LRRTM4 complexes reside separately at the synapses. GPC4 is secreted from astrocytes and interacts with presynaptic PTPδ. **(B)** Functions of *Drosophila* HSPGs (Dnrx, Dlp, and Sdc) in the NMJ synapses. Dnrx interacts with Dnlgn through HS chains (Zhang et al., 2018). Sdc promotes Dlar signaling, whereas Dlp suppresses it. Anterograde Wg and retrograde Gbb activities are regulated by fine structures of HS chains, presumably of Sdc and/or Dlp. Dlp regulates non-canonical BMP signaling through GluRIIA. Nrx, neurexin; Nlgn, neuroligin; LRRTM, leucine-rich repeat transmembrane neuronal protein; GPC, glypican; PTP, protein tyrosine phosphatase; GPR, G protein-coupled receptor; Dnrx, *Drosophila* neurexin; Dnlgn, *Drosophila* neuroligin; DFz2, *Drosophila* Frizzled 2; BMPR, BMP (bone morphogenetic protein) receptor; Sdc, syndecan; Dlp, Dally-like protein; Wg, Wingless; Gbb, Glass bottom boat.

**FIGURE 2:**
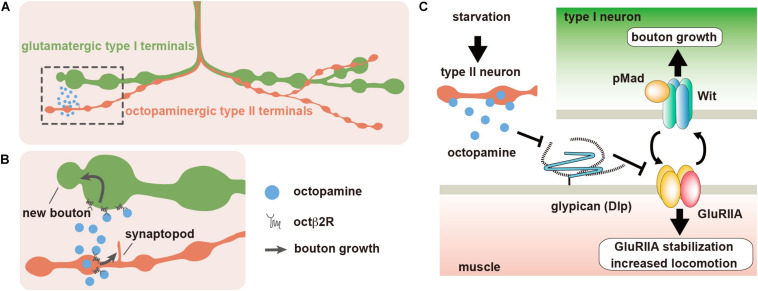
Dlp regulates synaptic plasticity at the *Drosophila* neuromuscular junction (NMJ). **(A)** The body wall muscles of *Drosophila* larva are innervated by type I glutamatergic motor neurons, which form synaptic boutons at the NMJs. Most larval body wall muscles are also innervated by octopaminergic type II neurons. **(B)** When larvae are placed under food deprivation conditions, octopamine is released from the type II boutons, and type II arbors rapidly extend new branches called synaptopods through the activation of Octβ2R octopamine autoreceptors. The acute acceleration of octopamine signaling by the growth of type II boutons leads to the rapid growth of type I boutons, which also express Octβ2R, and the increase of larval locomotor speed. **(C)** The expression of Dlp, which is a homolog of mammalian GPC4 and GPC6, is downregulated by octopamine signaling. Dlp suppresses the non-canonical BMP pathway that is composed of BMP receptor Wit and GluRIIA-containing iGluRs. Starvation-induced octopamine signaling downregulates Dlp expression, which leads to the activation of the non-canonical BMP signaling.

Synapses play essential roles in information processing in neural networks. During the development of neural networks, neurons extend axons and form synapses on specific target cells. After dynamic processes of synapse formation, synapses mature, and neural networks shift into full operation. Even after maturation of synapses, neural networks undergo local or large-scale changes called neural plasticity. Neural plasticity usually occurs in a neuronal activity-dependent manner at local synapses in response to internal and external stimuli, which serves as the basis for learning and memory (Oberman and Pascual-Leone, 2013). In recent years, it has been recognized that proteoglycans play critical roles in the development and plasticity of neural networks (Miyata and Kitagawa, 2017; Yu et al., 2018; Fawcett et al., 2019). Proteoglycans are cell surface and extracellular matrix (ECM) glycoproteins that carry sulfated glycosaminoglycans [chondroitin sulfate (CS), heparan sulfate (HS), and keratan sulfate] (Maeda et al., 2011). They are roughly classified into two groups: CS proteoglycans (CSPGs) and HS proteoglycans (HSPGs). It has been reported that CSPGs stabilize synapses and regulate neural plasticity and axonal regeneration (Miyata and Kitagawa, 2017). On the other hand, HSPGs, such as syndecans, have been considered as the regulators of synapse formation (Condomitti and de Wit, 2018). Quite recently, it was found that glypicans work as a component of synapse-organizing protein complexes. Synapse-organizing proteins play critical roles in the formation and function of synapses, controlling initial synaptic adhesion, maturation of synapses, as well as plasticity (Siddiqui and Craig, 2011; Takahashi and Craig, 2013; Um and Ko, 2013; Sudhof, 2017). Many of the synapse-organizing proteins form trans-synaptic complexes involving either presynaptic neurexins (Nrxs) or type IIa receptor protein tyrosine phosphatases (RPTPs) along with diverse postsynaptic binding partners. In this review article, we will first discuss the recent studies on the roles of glypicans in synapse formation and the function of vertebrate excitatory synapses, focusing on the interaction with synapse-organizing proteins (Figure 1A). Second, we will introduce the emerging evidence from fruit fly, *Drosophila*, studies showing that glypicans regulate synaptic and behavioral plasticity by the mechanism at least partially shared with that of vertebrate glypicans (Figures 1B, 2). Finally, we will discuss the animal model studies and human genetics implying the involvement of glypicans in neurodevelopmental and psychiatric disorders (Table 1).

**TABLE 1:** Dysregulation of glutamate receptors, behavioral defects, and mental disorders associated with GPC and HS-related genes.

	**Fruit fly**	**Mouse**	**Human**
GPC	*dlp:* • Dysregulation of GluRIIA-containing iGluRs (Kamimura et al., 2019) • Loss of starvation-induced synaptic and behavioral plasticity (Kamimura et al., 2019)	*GPC4*: • Dysregulation of GluA1 subunit of AMPARs (Allen et al., 2012) • ASD-like behaviors (Dowling and Allen, 2018)	*GPC1*: • Association with schizophrenia (Potkin et al., 2010) *GPC4*: • Association with ASD (Doan et al., 2016) *GPC6*: • Association with neuroticism and formal thought disorder (Calboli et al., 2010; Wang et al., 2012)
EXT	–	*Ext1*: • Dysregulation of GluA2 subunit of AMPARs (Irie et al., 2012) • ASD-like behaviors (Irie et al., 2012)	*Ext1*: • ASD with mild intellectual disability (Li et al., 2002)
Ndst	*sulfateless* (*sfl)*: • ASD-like behaviors (Hope et al., 2019)	–	*Ndst3*: • Association with schizophrenia and bipolar disorder (Lencz et al., 2013; Zhang et al., 2016)
Hs3st	–	–	*Hs3st-5*: • Association with ASD (Wang et al., 2009)

## HSPGs and HS Modification Enzymes

HSPGs are glycoproteins that possess long (50–200 sugar length) HS chains covalently attached to the serine residues of the core proteins (Sarrazin et al., 2011; Xu and Esko, 2014; Kamimura and Maeda, 2017). There are several types of HSPGs classified by their core protein structures. These include transmembrane-type syndecans, glycosylphosphatidylinositol (GPI)-anchored glypicans and secreted types, perlecan, and agrin. There are four syndecans (SDC1–4), six glypicans (GPC1–6), one perlecan, and one agrin in mice and humans (Godfrey et al., 1984; Ledbetter et al., 1985; Filmus et al., 1988; Marynen et al., 1989; Saunders et al., 1989; Carey et al., 1992; Kojima et al., 1992; Litwack et al., 1994; Stipp et al., 1994; Watanabe et al., 1995; Veugelers et al., 1997; Paine-Saunders et al., 1999). On the other hand, in *Drosophila*, only one syndecan (Sdc), two glypicans Dally-like (Dlp), and one perlecan (Trol) are identified (Spring et al., 1994; Nakato et al., 1995; Khare and Baumgartner, 2000; Voigt et al., 2002). HSPGs regulate various biological processes through the interaction with a wide range of molecules, such as growth factors and their receptors, cell adhesion molecules, ECM components, secreted proteases, lipoproteins, and bacterial and viral proteins (Sarrazin et al., 2011; Xu and Esko, 2014). Such versatile functions of HSPGs are thought to be regulated by the fine structures of HS.

In the biosynthetic processes, HS chains undergo tremendously complicated modifications, and their fine structures differ among tissues and cell types (Maeda, 2015). Biosynthesis of HS chains starts from the addition of a tetrasaccharide linkage region [xylose–galactose–galactose–glucuronic acid (GlcA)] to the core protein in the endoplasmic reticulum (ER). Xylose is transferred by xylosyltransferase to the specific serine residues on the core protein. Two galactose residues are transferred by galactosyltransferases I and II, and finally, GlcA is transferred by glucuronyltransferase I. Then, an *N*-acetylglucosamine (GlcNAc) residue is added to the linkage tetrasaccharide, which initiates the HS polymerization process in the Golgi apparatus. In the elongation processes, EXT family enzymes add alternating GlcA and GlcNAc residues. It should be noted that CS chains also use the same linkage tetrasaccharide. When an *N*-acetylgalactosamine (GalNAc) is added to the linkage tetrasaccharide instead of GlcNAc, elongation of CS chains begins (Maeda, 2015). During chain elongation, HS undergoes various modifications that include *N*-deacetylation and *N*-sulfation of GlcNAc by *N*-deacetylase/*N*-sulfotransferases (Ndsts), C5 epimerization of GlcA to iduronic acid (IdoA) by C5 epimerase, as well as variable *O*-sulfations at the C2 position of IdoA by 2-*O* sulfotransferase (Hs2st), at the C6 position of GlcNAc and *N*-sulfoglucosamine (GlcNS) units by 6-*O* sulfotransferases (Hs6sts), and at the C3 position of GlcNAc and GlcNS residues by 3-*O* sulfotransferases (Hs3sts). In addition to the sulfation and epimerization processes in the Golgi apparatus, extracellular 6-*O* endosulfatases (Sulfs) remove 6-*O* sulfate groups from specific subregions in HS chains. These modification processes do not occur uniformly along the HS chains, which leads to the generation of tremendous structural heterogeneity. Presumptive molecular mechanisms for this HS heterogeneity are as follows. First, HS modification enzymes show tissue-specific as well as cell type-specific expression patterns. Second, each enzyme shows particular enzymatic specificity for HS substrates. For example, Hs2st preferentially adds sulfate groups to IdoA rather than GlcA (Rong et al., 2001). Moreover, Hs6st-1 preferentially modifies GlcNS rather than GlcNAc, and Sulfs preferentially remove sulfate groups from tri-sulfated disaccharide units rather than di- and mono-sulfated disaccharides (Habuchi et al., 2000; Ai et al., 2003, 2006). Furthermore, several studies have shown that HS modification enzymes form the complexes in a specific combination called “gagosome” as observed between Ndst and EXT, C5 epimerase and Hs2st, and Hs2st and Hs6st (Pinhal et al., 2001; Esko and Selleck, 2002; Presto et al., 2008; Dejima et al., 2013). These findings suggest that interactions among HS biosynthetic enzymes affect the fine structures of HS. The distinct HS structures are considered to provide selective binding sites for distinct proteins. Indeed, several proteins, such as FGF-2 and antithrombin, require specific sulfate groups (2-*O* and 3-*O* sulfate groups, respectively) on HS (Habuchi et al., 1992; Turnbull et al., 1992; Shworak et al., 1997; Richard et al., 2009). In addition, HS chains of HSPGs are degraded into oligosaccharides at the cell surface and in the ECM by heparanase, regulating the activities of HSPG-binding proteins (Xu and Esko, 2014). Thus, the HS chains on HSPGs regulate the interactions with many ligand proteins in a structure-dependent manner, although the *in vivo* significance remains elusive.

## Functions of Vertebrate Glypicans at Synapses

### Glypican Interacts With Synaptogenic Protein, LRRTM4

Several studies in mice and rats revealed that glypicans (GPCs) interact with synapse-organizing proteins (Figure 1A). Among these, leucine-rich repeat transmembrane neuronal proteins, LRRTMs, are postsynaptic proteins that bind with presynaptic Nrxs at glutamatergic synapses (Siddiqui and Craig, 2011). The LRRTM family is composed of four members, LRRTM1–4, which share similar domain structures consisting of multiple extracellular leucine-rich repeats, a single transmembrane segment, and a cytoplasmic tail that binds to the postsynaptic protein PSD-95 (Lauren et al., 2003; Majercak et al., 2006; Francks et al., 2007; Haines and Rigby, 2007; Linhoff et al., 2009; Siddiqui et al., 2013; Soler-Llavina et al., 2013). All LRRTMs showed synaptogenic activity, which induced presynaptic differentiation in heterologous synapse formation assays (Lauren et al., 2003; Linhoff et al., 2009; Siddiqui et al., 2013; Soler-Llavina et al., 2013). Each LRRTM showed a specific expression pattern in the mouse brain. LRRTM1 and LRRTM2 were expressed in the CA1 region of the hippocampus, whereas LRRTM3 and LRRTM4 were expressed in the dentate gyrus (DG) (Lauren et al., 2003; Linhoff et al., 2009; Siddiqui et al., 2010). Each knockout mouse model for *LRRTM1*, *LRRTM3*, and *LRRTM4* genes showed significant but only small decreases in synapse density and synaptic transmission (Takashima et al., 2011; Siddiqui et al., 2013; Um et al., 2016). This may be due to the functional redundancy among LRRTM isoforms.

Two independent studies identified GPCs as the ligands for postsynaptic LRRTM4 (de Wit et al., 2013; Siddiqui et al., 2013). By a proteomic approach, de Wit et al. (2013) analyzed LRRTM-interacting proteins using rat whole-brain homogenates and found that the major surface protein bound with LRRTM4 was GPC4, whereas the most abundant proteins bound to LRRTM2 were Nrxs. Pulldown assays showed that LRRTM4 also bound to Nrxs; however, LRRTM4 did not simultaneously bind to GPC4 and Nrxs. Interaction between LRRTM4 and GPC4 was HS-dependent because the addition of free HS chains or digestion of HS by heparitinases suppressed the interactions. Immunohistochemical analyses showed that GPC4 and LRRTM4 were localized at the pre- and postsynaptic membrane of excitatory synapses in the rat brain, respectively. GPC4 expressed on HEK293T cells induced clustering of LRRTM4 on the membrane of co-cultured hippocampal neurons and *vice versa*, indicating that GPC4 and LRRTM4 interact *in trans*. Heparitinase treatment and siRNA-mediated knockdown experiments indicated that the synaptogenic activity of LRRTM4, but not of LRRTM2, was dependent on the HS portion of GPC4 on the presynaptic membrane. Similar results were also reported by Siddiqui et al. (2013), who used LRRTM4-Fc for the affinity purification of ligands and identified multiple isoforms of GPC.

A number of studies showed that LRRTMs are involved in AMPA receptor (AMPAR) trafficking and function (Sudhof, 2017). Simultaneous RNAi-mediated knockdown of LRRTM1 and LRRTM2 in the CA1 region of the mouse hippocampus reduced AMPAR-mediated synaptic currents and blocked long-term synaptic potentiation (LTP) (Soler-Llavina et al., 2011, 2013). Importantly, Nrx binding by LRRTM2 was responsible for LTP maintenance, and LRRTM1 and LRRTM2 maintained newly delivered AMPARs at synapses after LTP induction (Soler-Llavina et al., 2013). LRRTM4 was also identified as a component of AMPAR complexes and was required for LTP-induced synaptic surface trafficking of GluA1 subunits (Schwenk et al., 2012; Shanks et al., 2012; Siddiqui et al., 2013). As expected from the LRRTM4 distribution in the mouse hippocampus (see above), DG granule cells, but not CA1 pyramidal cells, in LRRTM4-KO mice exhibited reduction of excitatory synapse density and excitatory synaptic transmission (Siddiqui et al., 2013). Thus, LRRTM family members function at different subsets of excitatory synapses using distinct binding partners.

### Glypican Interacts With PTPσ *in cis*

By affinity chromatography using rat brain synaptosomes, Ko et al. (2015) found that presynaptic GPC4 also interacted with PTPσ in addition to LRRTM4 (Figure 1A). PTPσ is a member of the leukocyte common antigen-related (LAR) RPTP family and is one of the presynaptic synapse-organizing proteins (Yan et al., 1993; Takahashi et al., 2011). The LAR family is a type IIa subfamily of RPTPs, composed of three members in vertebrates, LAR, PTPσ, and PTPδ. These members contain immunoglobulin-like (Ig) and fibronectin III (FNIII) domains, a transmembrane segment, and two intracellular tyrosine phosphatase domains (Schaapveld et al., 1997; Johnson and Van Vactor, 2003; Chagnon et al., 2004). To date, several postsynaptic binding partners for LAR family members have been identified. For example, netrin-G ligand-3 (NGL-3) bound to LAR, PTPσ, and PTPδ (Woo et al., 2009; Kwon et al., 2010). TrkC bound selectively to PTPσ, whereas Slitrk3 selectively bound with PTPδ (Takahashi et al., 2011, 2012). Interleukin-1 receptor accessory protein-like 1 (IL1RAPL1) bound with PTPδ (Valnegri et al., 2011; Yoshida et al., 2011), and interleukin-1 receptor accessory protein (IL1RAcP) bound with LAR, PTPδ, and PTPσ (Yoshida et al., 2012; Takahashi and Craig, 2013).

GPC4–PTPσ interaction mainly occurred in the *cis*-configuration. Pulldown assay showed that PTPσ bound to LRRTM4 in the presence of GPC4, but not in the presence of GPC4-AAA, in which the HS attachment sites were mutated. These results suggested that PTPσ specifically forms complexes containing GPC4 and LRRTM4 through HS chains. Furthermore, siRNA-mediated knockdown of PTPσ decreased LRRTM4-mediated synaptogenic activity, which was restored by the expression of wild-type PTPσ but not by PTPσ-AAAA, in which the HS-binding site was mutated. These results suggested that the presynaptic PTPσ–GPC4 complex interacts with postsynaptic LRRTM4 to regulate synapse development, in which HS plays critical roles. They also demonstrated that knockdown of PTPσ, but not of LAR, decreased the frequency and amplitude of miniature excitatory postsynaptic currents (mEPSCs) in the culture of rat hippocampal neurons. These defects were fully rescued by the expression of wild-type PTPσ. However, PTPσ-AAAA only rescued the mEPSC amplitude and failed to recover the mEPSC frequency, suggesting that PTPσ regulates synaptic transmission in both HS-dependent and HS-independent manners. Future detailed studies are required to reveal the specific functions of the multiple ligands of PTPσ.

### Nrxs Function as HSPGs

Nrxs are type I membrane proteins that are the most well-known presynaptic organizers (Ushkaryov et al., 1992, 1994; Ushkaryov and Sudhof, 1993). Nrxs contain laminin/neurexin/sex-hormone-binding globulin (LNS) domains and three interspaced epidermal growth factor (EGF) domains (Missler and Sudhof, 1998; Reissner et al., 2013). In mammals, there are three *Nrx* genes (*Nrxn1*–*3*) (Sudhof, 2008; Reissner et al., 2013). Each of these *Nrx* genes contains two alternative promoters leading to the longer isoform, α-Nrx containing six LNS domains, and the shorter isoform, β-Nrx containing only one LNS domain, which is identical to the sixth LNS domain of α-Nrx (Sudhof, 2008; Reissner et al., 2013). α- and β-Nrxs interact with postsynaptic organizers, such as Nlgns and LRRTMs, through the common LNS domains (Ichtchenko et al., 1995; Ko et al., 2009; Sudhof, 2017). In addition to the α- and β-Nrx isoforms, Nrxs are alternatively spliced at six sites (referred to as SS#1 to SS#6), which may generate thousands of isoforms (Ullrich et al., 1995; Tabuchi and Sudhof, 2002; Treutlein et al., 2014; Sudhof, 2017). Thus, an extensive number of Nrx variants can be generated from the three different genes with two independent promoters and six alternative splice sites, leading to diversity and complexity in ligand-binding selectivity and synapse specificity (Reissner et al., 2013; Treutlein et al., 2014).

Zhang et al. (2018) showed that Nrxs function as HSPGs, adding further structural diversity to Nrxs (Figure 1A). To examine the function of the HS portion of Nrx in synaptic development and function, they performed siRNA-mediated knockdown of Nrx using cultured rat hippocampal neurons. As a result, the density of inhibitory synapses but not of excitatory synapses was reduced by Nrx knockdown. Furthermore, Nrx knockdown reduced the frequencies of mEPSC and miniature inhibitory postsynaptic current (mIPSC). These defects were rescued by RNAi-resistant Nrx but not HS-deficient Nrx, indicating that Nrx bearing HS chains are critical for the development of functional synapses. They next examined whether HS of Nrx was required for the interactions with its major postsynaptic ligands, Nlgns and LRRTM2, using a co-culture system. HS digestion by heparitinases blocked the activities of Nlgns and LRRTM2 to induce presynaptic differentiation. In addition, axonal Nrxs were locally aggregated at the contact sites on Nlgn1- or LRRTM2-expressing COS7 cells, but the HS-deficient Nrx was poorly aggregated. These results suggested that Nrxs interacted with both Nlgns and LRRTM2 through the HS chains during synapse formation. Consistent with this, Nlgns and LRRTM2 bound to heparin columns, and mutations in the HS-binding sites of Nlgns and LRRTM2 reduced their affinities for heparin and synapse-promoting activities. They also generated and characterized *Nrxn1*Δ*HS* mice, which carried point mutations that block HS modifications of Nrx. mEPSCs recorded from CA3 neurons showed reduction in both frequency and amplitude, and paired pulse ratio analyses of mossy fiber (MF)–CA3 synapses suggested a reduced probability of transmitter release in these mice. Furthermore, morphological analyses using serial block face scanning electron microscopy showed significant changes in the size and complexity of the synapses between MFs and CA3 thorny excrescence spines. Thus, the HS portion of Nrxs is required for Nrx functions in the synaptic development.

A recent study revealed that HS moiety of Nrxs not only bound to the postsynaptic Nlgns and LRRTMs, but they also formed complexes with the presynaptic synapse organizer, PTPσ (Figure 1A) (Han et al., 2020; Roppongi et al., 2020). LRRTM4 bound with Nrx through HS, but not with PTPσ. LRRTM4–Nrx–PTPσ complex was observed in the mouse synaptosomal lysates, and its formation was inhibited by heparitinase treatment. In addition, co-culture assay showed that both presynaptic Nrx and PTPσ were required for postsynaptic LRRTM4 to induce full presynaptic differentiation. Thus, HS of Nrx mediated the complex formation of LRRTM4–Nrx–PTPσ, which was required for synapse formation. Roppongi et al. (2020) also found that anti-LRRTM4 antibody co-immunoprecipitated GPC4, whereas anti-Nrx antibody co-immunoprecipitated LRRTM4 but not GPC4. This suggested that the LRRTM4–Nrx and LRRTM4–GPC4 complexes coexist independently. Furthermore, they generated knock-in mice for LRRTM4, in which the HS-binding site was mutated. In these mice, the levels of PTPσ and Nrx were reduced in the synaptosome fractions from DG. Immunofluorescence labeled synaptic puncta for VGlut1 were reduced in the molecular layer of DG, and transmission electron microscopic analyses showed that the density of excitatory synapses was reduced in the DG. Furthermore, the frequency and amplitude of mEPSCs and evoked excitatory transmission were reduced. Thus, the HS portion of Nrx mediates complex formation with LRRTM4 and PTPσ and plays critical roles in synapse formation and function. Functional relationship between the diversities of Nrx core proteins and the heterogeneity of attached HS chains needs further investigation.

Nrxs and GPC4 use common ligands, LRRTM4 and PTPσ, in which their ligand bindings are HS-dependent. Therefore, Nrxs and GPC4 may act competitively when they are expressed on the same synapses. Based on the RNAi experiments of hippocampal neurons, Roppongi et al. (2020) suggested that synaptogenic activities of LRRTM3 and LRRTM4 were primarily dependent on Nrxs rather than GPC4. Furthermore, they indicated that LRRTM3 and LRRTM4 expressed on COS7 cells recruited axonally expressed GPC5 in neurons, but this was not accompanied by presynaptic differentiation. At present, the functional relationship between Nrxs and GPCs is unclear, and future investigations are necessary to elucidate this relationship.

Khalaj et al. (2020) identified FAM19A, an “orphan” cytokine, as Nrx-binding protein from adult mouse brain. FAM19As bound with Nrxs through intermolecular disulfide bonds that were formed during intracellular transport. In the cultured hippocampal neurons, FAM19A decreased HS modification and *O*-glycosylation of Nrx without affecting its surface levels, suggesting that FAM19A regulates the posttranslational modification of Nrxs. Since FAM19A expression was restricted to the subsets of neurons and dependent on neuronal activity, HS modification of Nrx may be flexibly regulated in a neuronal activity-dependent and cell type-specific manner. Thus, HS modification of Nrxs and GPCs may be differentially controlled by FAM19A in the same neurons, leading to the differential regulation of their ligand-binding activities. Furthermore, FAM19A may influence the competitive activities of Nrxs and GPCs at the same synapses.

### Glypican 4 Interacts With GPR158 and Regulates Hippocampal MF–CA3 Synapse Formation

The hippocampal CA3 circuit plays critical roles in the encoding of memory (Rebola et al., 2017). GPC4 was localized on rat DG granule cell axons, the MFs, which form synapses on CA3 pyramidal neurons, but LRRTM4 was not detected in these neurons, suggesting the presence of other binding partners for GPC4 (de Wit et al., 2013; Siddiqui et al., 2013). To identify novel GPC4-binding proteins, Condomitti et al. (2018) used GPC4 as bait to perform pulldown assay on rat brain synaptosome extracts, and the interacting proteins were identified by tandem mass spectrometry. The identified major cell surface protein was GPR158, a G protein-coupled receptor (GPCR)-like orphan receptor with a large extracellular domain that shows homology to class C GPCRs (Orlandi et al., 2012). GPR158 was expressed on CA3 pyramidal neurons during MF–CA3 synaptogenesis, and the expression was restricted to the proximal segments of apical dendrites that receive MF inputs. Hippocampal neurons co-cultured with HEK293 cells expressing GPR158 showed presynaptic differentiation, which was impaired by HS digestion by heparitinases or shRNA-mediated knockdown of GPC4 in neurons. Thus, presynaptic GPC4 regulates GPR158-mediated presynaptic differentiation through the HS chains, and the binding partner of GPC4 depends on the postsynaptic targets (Figure 1A).

GPR158 KO mice showed impaired hippocampal-dependent learning as revealed by Morris water maze and novel object recognition tests (Khrimian et al., 2017). LTP at MF–CA3 synapses were also significantly impaired in these mice (Khrimian et al., 2017). Furthermore, Sutton et al. (2018) revealed that GPR158 is involved in stress-responsive behaviors and depression *via* the modulation of AMPAR activity. Future studies are necessary to investigate the roles of GPR158–GPC4 interaction in learning, stress-responsive behaviors, and depression.

### Glypicans Secreted by Astrocytes Induce Synapse Formation

Previous studies have shown that astrocyte-conditioned medium (ACM) induced functional synapse formation between rat retinal ganglion cells (RGCs) (Ullian et al., 2001). Allen et al. (2012) identified GPC4 and GPC6 from ACM as inducers of functional synapses between RGCs (Figure 1A). Purified GPC4 increased the frequency and amplitude of mEPSCs and the synapse numbers on RGCs. The increased synaptic activities were due to upregulation of cell surface levels of GluA1-containing AMPARs. They also showed that GPC6, which is most homologous to GPC4 among GPC family members, also recruited GluA1 to the cell surface and induced synapse formation on RGCs. In the mouse brain, GPC4 mRNA was enriched in the hippocampus, and GPC6 was enriched in the cerebellum during postnatal development. Especially, GPC4 was expressed in the hippocampal astrocytes at the early postnatal periods (P6–P14), during which synaptogenesis actively occurred. Furthermore, GPC4 knockout mice showed decreases in mEPSC amplitude and synapse number and reduced recruitment of GluA1 in the developing hippocampus (Table 1). Thus, GPCs secreted by astrocytes play critical roles in the formation of excitatory synapses containing AMPARs.

Farhy-Tselnicker et al. (2017) found that mRNA for neuronal pentraxin 1 (NP1), an ∼50 kDa secreted glycoprotein, was specifically upregulated by GPC4 in RGC cultures. A previous study showed that NP1 bound with the extracellular region of AMPARs and stabilized them on dendritic surfaces, leading to the promotion of synapse formation (O’Brien et al., 1999). They also found that the addition of soluble GPC4 to RGCs increased extracellular NP1 levels, showing that GPC4 stimulated NP1 release. In addition, inhibition of the NP1–GluA1 interaction by adding the blocking antibody prevented GPC4-mediated synapse formation on RGCs, indicating that NP1–GluA1 interaction was necessary for GPC4 to induce synapse formation. They also showed that the effects of GPC4 on NP1, GluA1, and synapse formation were regulated by presynaptic PTPδ, a member of LAR family RPTP (Figure 1A). Blocking the PTPδ function in RGCs using a specific cell-permeant wedge domain-blocking peptide reduced the GPC4-mediated synaptogenesis, clustering of GluA1, and surface accumulation of NP1. Finally, mice lacking either GPC4 or PTPδ showed increased retention of NP1 in presynaptic terminals, decreased recruitment of GluA1 to the synapses, and decreased synapse number in the developing visual systems. These findings revealed a functional link between astrocytes and the presynaptic-to-postsynaptic signaling pathways and provided important mechanistic insights into the roles of astrocyte-secreted GPC4 in AMPAR-mediated synaptogenesis.

## Functions of *Drosophila* Glypican, DLP, at Synapses

### Dlp Interacts With Dlar, a Member of LAR Family RPTP During Synaptic Development

The synaptic functions of glypicans are not limited to the vertebrate nervous system. Recent studies have focused on the *Drosophila* larval neuromuscular junction (NMJ). The body wall muscles of *Drosophila* larva are innervated by type I glutamatergic motor neurons, which form synaptic boutons at the NMJ with a beads-on-a-string morphology (Figure 2A). At these synaptic boutons, postsynaptic responses to glutamate are mediated by tetrameric ionotropic glutamate receptors (iGluRs), which are homologous to mammalian AMPARs or kainate receptors (Littleton and Ganetzky, 2000). Furthermore, these synapses share many important structural features with vertebrate central nervous system (CNS) excitatory synapses including well-developed postsynaptic density. Therefore, *Drosophila* larval NMJ is regarded as a simple model of vertebrate brain excitatory synapses.

Johnson et al. (2006) showed that both syndecan (Sdc) and Dlp, a homolog of mammalian GPC4 and GPC6, play important roles in the glutamatergic type I bouton formation at the NMJ. In *sdc* mutants, the number of type I synaptic boutons was reduced; on the other hand, mutations in *dlp* did not affect the type I bouton number. However, the active zones in the presynaptic boutons were smaller but increased in number in the *dlp* mutants compared with those of wild types. They also examined the electrophysiological properties of the NMJs of *sdc* and *dlp* mutant animals and found that *dlp* but not *sdc* mutants showed an increase in the amplitude of the excitatory junctional potential (EJP) elicited by nerve stimulation, indicating that *dlp* is required for normal synaptic transmission. Immunohistochemical studies showed that both Sdc and Dlp were localized at the postsynaptic sites in the glutamatergic type I boutons (Johnson et al., 2006; Nguyen et al., 2016). These studies also examined the binding partners for these HSPGs. Among the candidates, they focused on Dlar, an RPTP that is homologous to vertebrate LAR. Both Sdc and Dlp bound with Dlar, but the affinity of Dlp was twice as strong as that of Sdc. The bindings of Dlp as well as Sdc to Dlar were HS-dependent, and the difference in affinity may be explained by the structural difference in HS chains attached to these HSPGs (Figure 1B).

Genetic analyses indicated that the synaptic phenotypes of the double mutants of *sdc* and *dlar* were indistinguishable from those of *dlar* single mutants. Furthermore, overexpression of *sdc* did not promote the bouton formation when combined with *dlar* mutant background. These results indicated that the synaptic growth-promoting activity of Sdc was dependent on Dlar. On the other hand, overexpression of *dlp* reduced the number of synaptic boutons, which was similar to the phenotype of *dlar* mutants. In contrast, overexpression of *dlar* increased the bouton number. These results suggested that Sdc promotes Dlar signaling, whereas Dlp suppresses it. Thus, Dlp and Sdc regulate Dlar signaling, demonstrating the evolutionary conserved roles of HSPG–RPTP interactions in synaptic development and function (Figures 1A,B).

### Roles of HS Modification in Synaptic Function of HSPGs

Since HS chains regulate HSPG–ligand interactions, it is expected that fine structures of HS regulate the development and function of *Drosophila* NMJ. Dani et al. (2012) screened transgenic RNAi strains for 130 glycan-related genes to identify the genes showing defects in synapse morphology and electrophysiological characteristics. They found that knockdown of *Hs6st* and *Sulf1* genes, which add or remove a sulfate group at the 6-*O* position on HS, respectively, caused defects of NMJ. RNAi-mediated knockdown of both *Hs6st* and *Sulf1* resulted in the increased synaptic bouton numbers at the NMJ. In contrast, electrophysiological analyses showed that *Hs6st* RNAi decreased the amplitude of evoked excitatory junctional current (eEJC), whereas *Sulf1* RNAi increased it. Wingless (Wg) and Glass bottom boat (Gbb), *Drosophila* homologs of Wnt and BMP, respectively, are well-characterized anterograde and retrograde *trans*-synaptic heparin-binding signaling molecules, respectively, regulating NMJ formation (Packard et al., 2002; McCabe et al., 2003). Dani et al. (2012) also investigated the possibility that Hs6st and Sulf1 regulated the signaling activities of Wg and Gbb at the NMJ. Both *Hs6st* and *Sulf1* mutants showed the accumulation of extracellular Wg and Gbb proteins at the NMJs. Interestingly, the activity of Wg signaling was increased in *Hs6st*, but decreased in *Sulf1* mutants. However, the activity of BMP signaling was increased in both *Hs6st* and *Sulf1* mutants. These results showed that Wg and Gbb signaling activities were differentially regulated by Hs6st and Sulf1 (Figure 1B). Importantly, *Hs6st* mutants showed decreased synaptic levels of Dlp, but increased levels of Sdc, whereas *Sulf1* mutants showed increases of both HSPGs. This result indicated that 6-*O* sulfation of HS affects the HSPG levels at the NMJ, playing important roles in synapse formation and function.

### Regulation of HSPG Localization by mRNA-Binding Translational Repressor, FMRP

Fragile X syndrome (FXS) is a genetic condition that causes a variety of neurodevelopmental disorders, such as intellectual disability and autism spectrum disorders (ASDs) (Giraud et al., 1976; Harvey et al., 1977; Merenstein et al., 1996). These disorders are caused by loss of the *fragile X mental retardation 1* (*FMR1*) gene product (FMRP), which is an mRNA-binding translational repressor (Verkerk et al., 1991; Ashley et al., 1993; Eichler et al., 1993; Laggerbauer et al., 2001; Li et al., 2001). Many conserved FMRP targets were identified from patients with FXS and in animal disease models (mice and *Drosophila*), but the full spectrum of FMRP targets has yet to be elucidated (Tessier and Broadie, 2012; Fernandez et al., 2013). Friedman et al. (2013) used the *Drosophila fmr1* (*dfmr1*) null mutants as an FXS disease model to screen for upregulated neural proteins and found that Dlp and Sdc protein levels were upregulated at the NMJs of *dfmr1* null animals. Importantly, *dfmr1* null synapses exhibited altered trans-synaptic signaling mediated by Wg and Jelly belly (Jeb), which is regulated by the endogenous lectin, Mind-the-Gap. To examine whether the synaptic defects of *dfmr1* mutants were caused by the altered Sdc and Dlp levels, the expression levels of these HSPGs were genetically reduced to the control levels in *dfmr1* null animals. Correction of HSPG levels restored the Wg and Jeb signaling as well as the synaptic architecture and transmission strength to the wild-type levels in *dfmr1* null animals. Taken together, these data suggested that FMRP negatively regulates Dlp and Sdc levels, controlling the trans-synaptic signaling during synaptogenesis.

### Matrix Metalloproteinases Regulate Localization and Function of Dlp

As described above, Allen et al. (2012) showed the importance of astrocyte-derived soluble GPCs in synaptogenesis. However, the mechanism of GPC release is unclear. Dear et al. (2016) revealed that matrix metalloproteinases regulate Dlp function at the *Drosophila* NMJ. In *Drosophila*, there is only one secreted MMP (Mmp1) and one GPI-anchored MMP (Mmp2) in contrast to the many MMPs in vertebrates (24 MMP genes in mice), providing an excellent model for elucidating MMP function. Mutations in either *mmp1* or *mmp2* increased the synaptic bouton number and elevated neurotransmission at the NMJ. Dlp protein level and Dlp-positive domain were strongly reduced in the NMJ boutons of *mmp1* mutants. In contrast, Dlp-positive domain was expanded in the *mmp2* mutants, indicating that Dlp distribution was reciprocally regulated by Mmp1 and Mmp2. Correction of Dlp distribution by overexpression or heterozygosity of *dlp* rescued the morphological and electrophysiological defects of *mmp1* or *mmp2* mutants, respectively. These results showed that MMPs regulate Dlp distribution at the NMJs to coordinate synapse development probably by regulating shedding of Dlp.

The association between MMP and Dlp was also demonstrated by Dear et al. (2017). At the *Drosophila* NMJ, acute neuronal stimulation by expression of temperature-dependent ion channel dTRPA1 (transient receptor potential cation channel A1) or high K^+^ stimulation induced presynaptic bouton formation. Synaptic Mmp1 protein levels were increased by such stimulation, and *mmp1* was required for this activity-dependent synaptic bouton formation. They also found that neuronal stimulation increased Dlp levels within the same synaptic subdomains as Mmp1. Reduction of *dlp* lowered Mmp1 levels, and overexpression of *dlp* increased Mmp1 levels; however, overexpression of HS-deficient *dlp* did not affect Mmp1 levels. This suggested that Dlp promotes synaptic localization of Mmp1 through the HS chain. In addition, they found that synaptic induction of Mmp1 by neuronal activity required *dlp*. These results showed that Dlp regulates basal and activity-dependent Mmp1 localization at the NMJ synapses. Furthermore, it was shown that there was no change in synaptic Mmp1 levels after neuronal stimulation in *dfmr1* null mutants, in which Dlp expression was upregulated at the NMJs. Reducing Dlp levels by heterozygosity of *dlp* fully restored the activity-induced Mmp1 increase in *dfmr1* null animals, suggesting that Dlp misregulation in FXS model animals mediates the Mmp1 dysfunction and the defects of activity-dependent synaptogenesis.

### Dlp Regulates Experience-Dependent Synaptic and Behavioral Plasticity

Animals in their natural habitat are often exposed to severe environmental changes, which may lead to starvation. Animals must change their behaviors to overcome such difficulties and survive. *Drosophila* larvae show this type of behavioral plasticity under experimental conditions. They are usually raised in culture vials containing food slurry and spend most of the time feeding inside the slurry. However, when larvae are transferred from culture vials to a food-free plate, they immediately initiate rapid locomotion to search for food. It is known that the type I bouton outgrowth occurs at the NMJ during rapid locomotion, which is a typical example of experience-dependent synaptic plasticity (Figure 2B) (Koon et al., 2011). In addition to the glutamatergic type I synaptic boutons, there are also octopaminergic type II synaptic boutons at the NMJ of most larval body wall muscles (Figure 2A). Octopamine is the invertebrate counterpart of noradrenaline, which regulates various behaviors, such as foraging, sleep, and aggression (Yang et al., 2015; Pereira and Murthy, 2017). This type II modulatory innervation regulates the type I bouton growth during locomotion under starved conditions. After food deprivation, octopaminergic type II axons rapidly extend new branches by the activation of its own Octβ2R octopamine receptors, constructing the positive feedback loop that promotes the growth of type II boutons (Figure 2B). Since Octβ2R is also expressed on type I axons, the acute activation of octopamine signaling by this positive feedback loop leads to rapid increases in type I boutons and locomotor activity (Figure 2B). We recently revealed that Dlp plays critical roles in these processes (Kamimura et al., 2019) (Table 1).

We first examined the postsynaptic Dlp levels at the type I boutons under starved conditions. We found that postsynaptic Dlp protein levels decreased during the first 4 h after food deprivation. This change in Dlp expression was not observed in the animals that were defective of octopaminergic signaling, showing that octopamine regulates postsynaptic Dlp levels during starvation. Under normal rearing conditions (fed conditions), type I bouton number and locomotor speed were increased in the *dlp*-underexpressing animals compared with wild-type animals, indicating that Dlp negatively regulates type I bouton growth and locomotor speed. These observations suggested that octopamine signaling negatively regulates Dlp expression, which in turn promotes type I bouton growth and locomotion during starvation. Indeed, the number of type I boutons and the locomotor speed remained at high levels in the heterozygous animals and muscle-specific knockdown animals for *dlp* before and after food deprivation, and the starvation-induced changes were not observed. Thus, postsynaptic Dlp levels determine the type I bouton number and locomotor speed, and octopamine-induced decreases in Dlp expression lead to increase in this number and speed during starvation.

We next performed a mechanistic study. Electrophysiological analyses of the body wall muscles of control larvae showed that the amplitude of evoked EJP (eEJP) increased, whereas the amplitude and frequency of miniature EJP (mEJP) decreased after food deprivation; thus, the quantal content (the number of synaptic vesicles released with each stimulus) increased in control animals. However, in *dlp*-knockdown larvae, the eEJP amplitude, mEJP frequency, and quantal content did not significantly change even after food deprivation, although the mEJP amplitude slightly increased. These results indicated that Dlp is required for the changes in synaptic transmission after food deprivation. The changes in electrophysiological properties during starvation suggested the importance of postsynaptic iGluRs. These iGluRs are composed of three essential subunits (GluRIIC, GluRIID, and GluRIIE) and a GluRIIA (type A iGluR) or GluRIIB subunit (type B iGluR) (Qin et al., 2005). Previous studies revealed that the regulation of postsynaptic GluRIIA levels plays important roles in physiological properties of the NMJ (Sigrist et al., 2002, 2003). Consistent with this, we found that knockdown of *GluRIIA* prevented the starvation-induced behavioral and synaptic plasticity. Importantly, postsynaptic levels of GluRIIA increased after food deprivation in wild-type animals, which was inversely correlated with Dlp expression. In addition, postsynaptic GluRIIA levels were already increased in the *dlp*-underexpressing animals under fed conditions, and these levels did not further change even after food deprivation. This suggested that Dlp negatively regulates the postsynaptic levels of GluRIIA, and therefore, those of type A iGluRs (Figure 2C).

Sulkowski et al. (2014, 2016) identified postsynaptic type A iGluRs as a novel ligand for presynaptic Wit, a type II BMP receptor. This non-canonical BMP signaling does not require a well-known BMP ligand, Gbb, but depends on Mad, a downstream transducer of BMP signaling. In this signaling pathway, type A iGluRs activate Wit and promote the accumulation of phosphorylated Mad (pMad) at the presynaptic active zone. Presynaptic pMads then recruit type A iGluRs at the postsynaptic sites, forming a positive feedback loop that leads to the accumulation of postsynaptic type A iGluRs (Figure 2C). In order to study the involvement of BMP signaling in Dlp-mediated synaptic and behavioral plasticity, we first examined *wit* and *gbb* mutant larvae. As a result, we found that mutations in *wit* but not *gbb* prevented starvation-induced increases in type I boutons and locomotion speed as observed in *GluRIIA*-knockdown animals and *dlp*-underexpressing animals. As expected, since starvation increased postsynaptic GluRIIA levels, presynaptic pMad levels increased after food deprivation in control animals. In contrast, presynaptic pMad levels were already increased in the *dlp*-underexpressing animals under fed conditions, and these levels did not further change even after food deprivation, as observed in the postsynaptic GluRIIA levels of these animals. This suggested that Dlp downregulates either presynaptic Wit activity or postsynaptic GluRIIA levels, which in turn suppresses non-canonical BMP signaling. Under starved conditions, octopamine-induced decrease of postsynaptic Dlp leads to the disinhibition of non-canonical BMP signaling that results in the accumulation of postsynaptic GluRIIA and type I bouton growth (Figure 2C).

To determine the steps of Dlp function in the non-canonical BMP signaling, we examined the postsynaptic levels of GluRIIA in *wit* null mutants and *wit* null animals with *dlp* RNAi. As a result, postsynaptic GluRIIA levels in *wit* mutants with *dlp* RNAi were higher than those in *wit* mutants, indicating that Dlp downregulates GluRIIA levels in the absence of *wit*. Thus, these results demonstrated that Dlp regulates synaptic and behavioral plasticity by adjusting the postsynaptic levels of GluRIIA in response to octopamine signaling (Figure 2C).

Dlp maintains an appropriate level of non-canonical BMP signaling at the NMJ under normal conditions. Acute environmental changes (food deprivation in this case) decrease the expression levels of Dlp in response to octopamine signaling, leading to the rapid activation of non-canonical BMP signaling and changes of synaptic number and behavior. Behavioral plasticity and flexibility are critically important for animals and humans to adapt to the environment. Lack of behavioral flexibility and stereotypic behaviors are often found in people with ASD, intellectual disability, and schizophrenia. NMJs of *dlp* mutants may be useful as a simple model for these disorders (Table 1).

### Animal Models Suggest Involvement of Glypicans and HS in ASDS

ASD is characterized by three core deficits: impaired reciprocal social interaction, impaired communication, and restricted, repetitive, and stereotyped patterns of behaviors (Silverman et al., 2010). Several studies indicated that defects in GPCs and HS synthesis lead to ASD-like phenotypes in animal models (Table 1) (Irie et al., 2012; Dowling and Allen, 2018; Hope et al., 2019). Irie et al. (2012) generated conditional knockout mice for Ext1, an enzyme essential for HS chain elongation (Ext1^CKO^ mice), in which *Ext1* was selectively removed from postnatal glutamatergic neurons in the forebrain. These Ext1^CKO^ mice grew normally and did not show apparent defects in brain morphology, such as neuronal lamination pattern and fiber tracts. These mice also exhibited no detectable abnormalities in motor functions, reflexes, olfaction, and vision; however, they showed reduced nest-building activity, which is a phenotype considered to be implicated in ASD. Detailed analyses showed that Ext1^CKO^ mice recapitulated three core deficits of ASD. First, social interaction skills were examined using the separation–reunion test, resident–intruder test, and social dominance tube test. For all these analyses, Ext1^CKO^ mice showed significant impairment in social interaction. Second, they analyzed linguistic communication. When wild-type mice were challenged by female odor, they emitted a series of complex ultrasonic vocalizations (USVs). In contrast, USVs emitted by Ext1^CKO^ mice were weak and simple. Third, stereotypic and repetitive behaviors were detected in Ext1^CKO^ mice using a hole-board test. Wild-type mice explored different holes in a random or successive manner, whereas Ext1^CKO^ mice showed repetitive head-dips into the same hole. In addition, Ext1^CKO^ mice showed reduced fear of height and open spaces, as well as sensory hypersensitivity to thermal stimuli. These phenotypes may also be relevant to ASD because a lack of fear in response to danger and odd responses to sensory stimuli are occasionally observed in individuals with ASD.

In Ext1^CKO^ mice, neuronal activities in response to social stimulation were attenuated in the basolateral and medial amygdala as revealed by c-Fos immunostaining. Electrophysiological analyses of the pyramidal neurons in the basolateral amygdala showed that the frequency and amplitude of AMPAR-mediated mEPSCs were reduced in Ext1^CKO^ mice. Consistently, the surface expression of the GluA2 subunit of AMPAR was reduced by 41% in the synapses of primary cultured neurons from Ext1^CKO^ mice. Thus, HS is critical for the functions of AMPARs, and deficiency of HS causes abnormal behaviors characteristic for ASD.

As described above, astrocyte-derived soluble GPC4 induces the formation of excitatory synapses by increasing the levels of GluA1-containing AMPARs. On the other hand, presynaptic GPC4 expressed in subsets of neurons acts as a ligand for the synapse-organizing proteins on the postsynaptic neurons, leading to presynaptic differentiation. Astrocytic expression of GPC4 is high during juvenile stages, and neuronal expression increases with maturation. Dowling and Allen (2018) examined the behavior of GPC4 KO mice at juvenile and adult ages. Open field test showed that the total distance moved and the average velocity were significantly increased in juvenile (P14) GPC4 KO mice; however, this hyperactivity was lost at P21, when defects in synaptic strength in the hippocampus were somewhat improved. Thus, the hyperactivity of GPC4 KO mice was transient, matching the time period when decrease in synaptic GluA1 occurred in these KO mice probably because of the loss of astrocyte-derived GPC4. Adult GPC4 KO mice at 3 months old did not show any defects in hearing, vision, smell, and grip strength. Furthermore, there was no significant difference between the wild-type and GPC4 KO mice in open field tests, Y-maze spontaneous alteration tests, and light–dark tests, showing that mobility, working memory, and anxiety-related behavior were intact in these KO mice. On the other hand, these GPC4 KO mice showed deficits in social behaviors. They performed a social novelty test, where a mouse chooses between a chamber containing a familiar mouse and a chamber containing a novel mouse. Wild-type mice spent more time with a novel mouse than a familiar mouse, but GPC4 KO mice showed no preference for either mouse, indicating a deficient behavior in social novelty. Thus, the defects of adult GPC4 KO mice were partially related to ASD. Differences in the major origin of GPC4 (astrocytes or neurons) and in the affected neural circuits may explain these age-dependent phenotypes of GPC4 KO mice.

Recently, *Drosophila* models have been used for identification of genes relevant to ASD. Hope et al. (2019) assayed a set of fly behaviors that may be analogous to those of ASD patients. Social reciprocity was examined by the social spacing test, which utilizes the natural tendency of flies to move upward in the vertically oriented chambers and settle into a spacing of ∼1.5 fly length apart. To study social communication, they focused on the mating process. Male flies generate a “love song” by vibration of their wings, and female flies will mate if they respond to this song. Thus, latency to mating during courtship behavior was measured to quantify expressive and receptive communication. Repetitive behaviors/restricted interests were assayed using a grooming behavior test. Using these assays, Hope et al. (2019) screened the *Drosophila* genetic reference panel (DGRP) lines, which have been derived from a natural population and already sequenced (Mackay and Huang, 2018). Forty DGRP lines were selected at random from the collection, and each line was tested for the above behaviors. They identified many variants [single nucleotide polymorphisms (SNPs), indels, or insertions] in several ASD-related genes, such as Nlgn2 and Nrx4, from each behavioral assay, but only one gene was associated with all behaviors tested: *sulfateless* (*sfl*), which encodes for Ndst (Lin and Perrimon, 1999) (Table 1). To confirm the *sfl* function in these behaviors, RNAi-mediated knockdown of *sfl* in neurons was performed. As a result, *sfl*-RNAi flies showed longer copulation latencies, increase in social spacing, and decrease in the number and time of grooming. These results indicated the importance of HSPGs in ASD-associated behaviors and the advantages of using *Drosophila* to understand the etiology of ASD.

## Glypicans in ASD and Other Psychiatric Disorders

Consistent with the results from animal models, it was reported that GPCs and HS were indeed associated with ASD. Doan et al. (2016) focused on the roles of human accelerated regions (HARs), genomic loci that are largely conserved among mammals but are strikingly different in humans, which have been suggested to reflect the potential roles in the evolution of human-specific traits, such as language, civilization, and complex society. They investigated the mutational landscape of HARs by comparing *de novo* copy number variations and biallelic point mutations in individuals with ASD and healthy controls. From this analysis, they identified two unrelated families possessing mutations in the same HAR within an intron of GPC4 (Table 1). Luciferase analyses of the control and mutant HAR sequences using the GPC4 promoter showed that both mutations reduced regulatory activity by 20–25%. These results suggested that the altered expression of GPC4 leads to ASD risk.

Pearson et al. (2013) examined the distribution of *N*-sulfated HS in postmortem brain tissues of individuals with autism using immunofluorescence techniques and found that HS was reduced in the subventricular zone of the lateral ventricles, which is a critical neurogenic niche found to be associated with ASD (Kotagiri et al., 2014). Li et al. (2002) reported two boys from separate families with mutations in the *Ext1* gene (Table 1). These patients presented hereditary multiple exostoses (an autosomal dominant inherited bone disorder) and autism associated with mild intellectual disability. Furthermore, Wang et al. (2009) performed genetic analyses of a large number of ASD cases and families of European ancestry. They identified a SNP associated with ASD near the gene encoding Hs3st-5 (Table 1). These results suggest the possibility that defects of HS synthesis influence the ligand-binding activities of GPC4 and/or Nrx, causing synaptic dysfunction and ASD.

In addition to ASD, several studies reported that GPCs and HS were associated with personality traits as well as neurodevelopmental and psychiatric disorders (Table 1). Neuroticism is a moderately heritable personality trait involving a long-term tendency to be a negative emotional state, which is thought to be a risk factor for the development of depression, anxiety disorders, and dementia. Calboli et al. (2010) performed a genome-wide association study (GWAS) of neuroticism in 2,235 participants by using the Eysenck Personality Questionnaire (EPQ). When measured by the EPQ, neuroticism was shown to be relatively stable over 20 years, although there was some evidence of a moderate decline in older ages. SNP by age interaction analyses revealed multiple associations with the *GPC6* gene. The strongest signal was at a genotyped SNP, rs9561329, and the EPQ score increased with age for these alleles, but decreased for non-carriers. GPC6 was also associated with formal thought disorder (FTD) in schizophrenia (Wang et al., 2012). FTD is one of the main symptoms of schizophrenia, but there are few studies investigating genetic variants in FTD. Wang et al. (2012) performed a meta-analysis of the two GWAS data from the Affymetrix Genome-Wide Human SNP array 6.0 to identify genetic variants influencing FTD in schizophrenia using European–American samples. As a result, four genes associated with FTD in schizophrenia were identified including the *GPC6* gene.

Potkin et al. (2010) attempted to identify the gene regulatory networks that are associated with schizophrenia using the brain imaging integrated with genetic data from GWAS. They measured the Blood Oxygenation Level Dependent (BOLD) functional MRI (fMRI) signals in the dorsolateral prefrontal cortex (DLPFC) during the working memory task as a quantitative trait phenotype of schizophrenia, in which schizophrenics showed more BOLD activation in the DLPFC than healthy controls. They identified GPC1 as a candidate risk factor for schizophrenia and proposed a model, in which epistatic interactions between GPC1 and FGF17 during brain development are involved in the etiology of schizophrenia (Table 1).

Lencz et al. (2013) performed a GWAS in an ethnically homogeneous cohort of Ashkenazi Jewish patients with schizophrenia (904 cases) and identified rs11098403 as a top SNP. This significant risk SNP was replicated in 11 independent schizophrenia or bipolar disorder cohorts of varying ethnicities. They also examined the function of rs11098403 by testing the effect of this variant on gene expression in postmortem brain tissue and by performing *in vitro* and *in silico* experiments. These analyses revealed a potential role of rs11098403 in the expression of the neighboring gene, *Ndst3* (Table 1). Importantly, similar results were also obtained from another study focusing on the Han Chinese population (Zhang et al., 2016). These studies indicated the importance of HS for understanding the pathophysiological mechanism of schizophrenia and bipolar disorder.

## Conclusion and Future Directions

**FIGURE 3:**
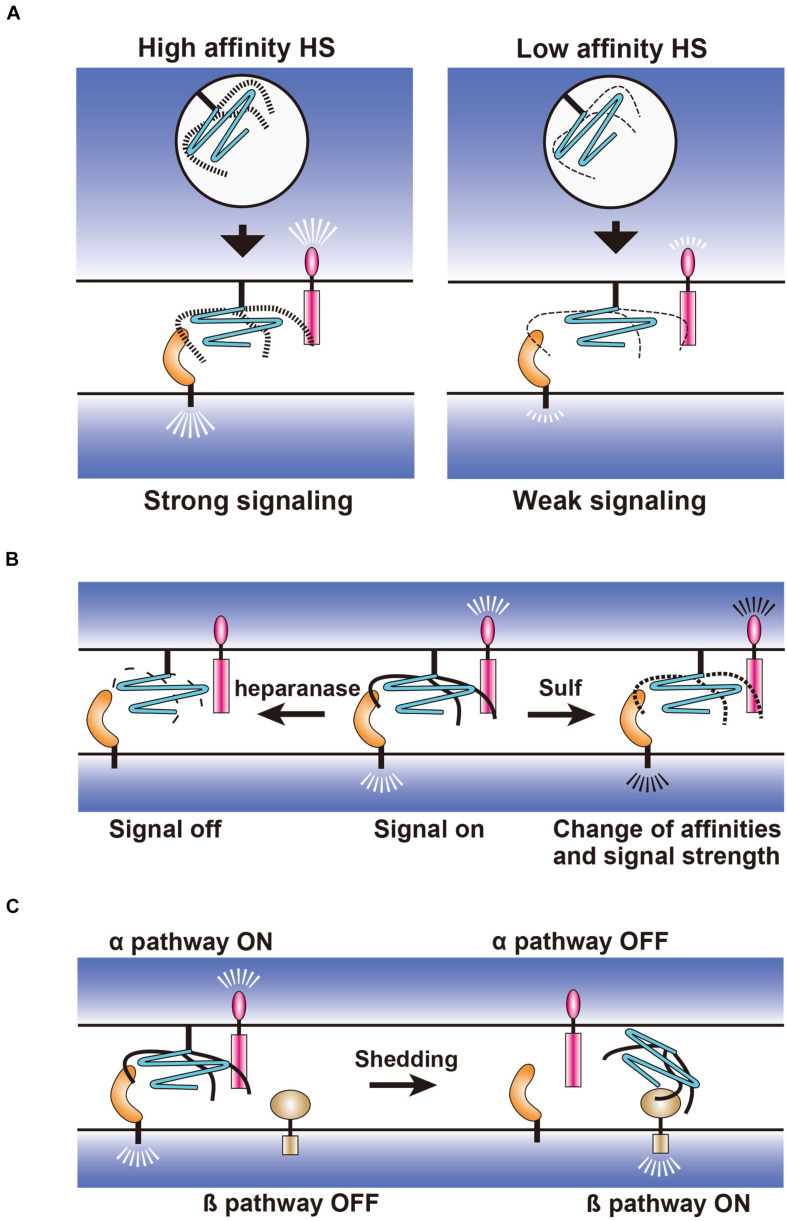
Hypothetical models for the regulation of synapse-organizing proteins by glypicans. **(A)** Fine structures of HS generated during biosynthetic processes may regulate the affinity strength between the synapse-organizing proteins affecting their signal strengths. **(B)** Extracellular regulation of HS structures by heparanase and 6-*O* endosulfatase (Sulf). Fragmentation of HS by heparanase and 6-*O* desulfation by Sulf may turn the signal off and change the signal strength of synapse-organizing proteins, respectively. **(C)** Effects of the glypican shedding on the synapse-organizing proteins. Shed glypicans may not activate synapse-organizing proteins (α pathway), but instead activate an alternative signaling pathway (β pathway).

GPCs are emerging as novel members of synapse-organizing proteins, which regulate synapse formation and synaptic function of the glutamatergic neurons. In the vertebrate brain excitatory synapses, GPC4 and Nrxs work as presynaptic ligands for postsynaptic LRRTM4, in which HS chains play critical roles. Furthermore, presynaptic PTPσ also interacts with GPC4 and Nrxs through HS chains. Currently, it is unclear why the synapse-organizing proteins use HS chains in addition to the protein domains. One possibility is that HS regulates the signal strength of synapse-organizing proteins. The structural variations of HS that are generated by HS-modifying enzymes may regulate the affinity strength between the synapse-organizing proteins (Figure 3). This expands the range of affinity strengths that cannot be achieved by simple protein–protein interactions. Such flexible regulation may diversify the strength of signaling between pre- and post-synaptic regions, which is the prerequisite for the differentiation of specific and diverse synapses. For example, if both GPC4 and Nrxs bear high affinity HS chains, both of their signaling would be strong. On the other hand, if one has high affinity HS chains and the other has low affinity chains, the signaling of the former would be strong, but that of the latter would be weak. Thus, the signal strengths of synapse-organizing proteins may be flexibly regulated by the structural variation of HS chains in many different contexts. The other possibility is that the interactions between multiple pre- and postsynaptic synapse-organizing proteins are simultaneously regulated by the HS chains with particular structures because the multiple and long HS chains of HSPGs can interact with multiple proteins at the same time. It is speculated that such global regulation is more easily achieved by changing the HS structures than by changing the expression level of each synapse-organizing protein. Thus, HSPGs may function as a hub that determines the synaptic specificities and overall activities of synapse-organizing proteins.

Structural changes of HS may be achieved through the regulation of HS biosynthetic enzymes in the Golgi apparatus (Figure 3A) or through the extracellular desulfation by Sulfs and fragmentation by heparanase (Figure 3B) (Sarrazin et al., 2011). Expressions of each HS sulfotransferase and C5 epimerase are differentially regulated in the distinct types of neurons during synaptic differentiation (Yabe et al., 2005). In addition, neuronal expression levels of these HS biosynthetic enzymes might be altered in a neuronal activity- and experience-dependent manner. Thus, neurons may express HS chains with unique structures at specific developmental stages and in particular environments, which influence the affinity between HS and their binding proteins. The Golgi-resident HS biosynthetic enzymes only act cell-autonomously, whereas Sulfs and heparanase modify HS extracellularly and thus may act non-cell-autonomously (Figures 3A,B). Therefore, these extracellular enzymes may have a broader impact on HSPG-related signaling pathways and synapses than Golgi-resident enzymes. Another important regulation is the shedding of GPCs, which is released from the plasma membrane by the cleavage of GPI anchors or core proteins (Figure 3C). It is considered that the released GPCs cannot activate synapse-organizing proteins, but may activate an alternative signaling pathway instead. It will be important to study these HSPG-related enzymes during synapse formation and synaptic plasticity. Genetic interaction analyses of HSPG-related enzyme and HSPG core protein genes will reveal how HSPG modifications regulate the synaptic function of specific HSPGs. Such experiments may be easily performed using *Drosophila* because this animal model has only one or two genes for each HSPG and HS synthetic/modifying enzyme (Kamimura and Maeda, 2017). It is also informative to examine the changes in the localization of HSPGs and HS-modifying enzymes after neuronal stimulation by *in vitro* and *in vivo* imaging.

The glutamatergic synapses of *Drosophila* larval NMJ show functional and structural similarities to those of vertebrate brains. It is striking that both systems utilize common HSPGs, GPCs, and Nrxs. Dnrx and Dlp interact with Dnlgn and Dlar, respectively, as vertebrate Nrx and GPC4 interact with Nlgn and PTPσ, respectively. It is important to investigate whether the other synapse-organizing proteins also interact with Dnrx and Dlp. Another important similarity is that both GPC4 and Dlp regulate the postsynaptic expression levels of iGluRs, GluA1-containing AMPARs, and GluRIIA-containing iGluRs, respectively (Table 1). *Drosophila* iGluRs are homologous to mammalian AMPARs (Littleton and Ganetzky, 2000), which support the validity of *Drosophila* NMJs as a model of mammalian excitatory synapses. The study of Dlp functions at *Drosophila* NMJ would provide important hints on the dynamic regulation of AMPARs at the mammalian brain excitatory synapses.

At the *Drosophila* larval NMJ, Dlp expression is regulated by the octopaminergic system, which modulates various behaviors, such as foraging, sleep, and aggression. In vertebrates, analogous functions are performed by the noradrenergic system. In each abdominal hemisegment of the *Drosophila* larva, there are 30 body wall muscles, which are innervated by glutamatergic type Ib neurons in an approximately 1:1 manner. In contrast, only three type II octopaminergic neurons bilaterally innervate the body wall muscles in each abdominal segment (Hoang and Chiba, 2001). This suggests that type II octopaminergic neurons globally regulate the properties of type I terminals, such as synaptic excitability, thereby coordinating the activities of the body wall musculature. This situation is similar to the mammalian brain noradrenergic system. Noradrenaline-producing neurons mainly cluster in the locus coeruleus, which is located in the pons with global projections throughout the brain (Schwarz et al., 2015). Thus, it will be important to study whether GPC expressions in mammalian brains are regulated by the noradrenergic system. If GPC functions are regulated by the noradrenergic system, for example, by stimulation of GPC4 secretion from astrocytes, then the activities of excitatory synapses may be globally enhanced all at once, leading to acute behavioral changes.

GPCs regulate multiple signaling pathways at the glutamatergic synapses, and it is considered that the external stimuli and animal experiences change the expression levels of GPCs, leading to the modulation of the activities of these pathways. When such appropriate control of GPC expression is lost, flexible behaviors are expected to be disturbed. Indeed, defects in GPC and HS synthetic/modifying enzyme genes cause neurodevelopmental and psychiatric disorders, such as ASD and schizophrenia (Table 1). Therefore, elucidating the detailed mechanisms of GPC actions on the glutamatergic synapses will provide the molecular basis of these disorders and effective therapies.

## Author Contributions

KK and NM wrote the manuscript. Both authors contributed to the article and approved the submitted version.

## Conflict of Interest

The authors declare that the research was conducted in the absence of any commercial or financial relationships that could be construed as a potential conflict of interest.
